# Characterisation of Monovarietal Olive Oils Obtained from Croatian *cvs.* Drobnica and Buza during the Ripening Period

**DOI:** 10.3390/foods7110188

**Published:** 2018-11-13

**Authors:** Jasminka Giacometti, Čedomila Milin, Fabio Giacometti, Zlatko Ciganj

**Affiliations:** 1 Department of Biotechnology, University of Rijeka, Radmile Matejčić 2, HR-51000 Rijeka, Croatia; 2 Faculty of Medicine, Department of Chemistry and Biochemistry, University of Rijeka, Braće Branchetta 20, HR-51000 Rijeka, Croatia; cedomila.milin@medri.uniri.hr; 3 Ekoplus, d.o.o., Pogled 2/4, HR-51216 Viškovo, Croatia; fabio.giacometti@ekoplus.hr; 4 INA Refinery Urinj, HR-51000 Rijeka, Croatia; zlatko.ciganj@gmail.com

**Keywords:** buza olive oil, drobnica olive oil, ripening, saponifiables and unsaponifiables, olive oil phenolics, trace minerals

## Abstract

The aim of this study was the monitoring of the chemical composition of olive oil at different ripening stages to determine the appropriate harvesting time during any given crop season in the northern Adriatic region. For this purpose, from September to November, two Croatian olive cultivars (Drobnica and Buza) were taken from two different olive orchards and for the respective olive oils, prepared on a laboratory scale, the major saponifiable, unsaponifiable and phenolic compounds were determined. Based on the chemical analyses performed, the optimal harvesting time has been set in October for both cultivars. Buza had a higher oleic acid, but lower total sterols, squalene and total alkanols. Compared to the local cultivars, the studied cultivars had a high total phenolic content and antioxidant activity. The antioxidant activity and concentrations of total phenols correlated with α-tocopherol in oil samples taken during the ripening progress. Finally, trace minerals detected in Buza and Drobnica oil differed, which can be an indicator of oxidative stability and authenticity of oils.

## 1. Introduction

The olive tree (*Olea europaea* L.) is one of the most widely, traditionally cultivated and economically relevant plants in Mediterranean countries. As the main component of the Mediterranean diet, olive oil has numerous nutritional as well as generally positive effects on human health. Therefore, it is important to produce olive oil with the best chemical composition, nutritive characteristics and beneficial effect on human health [1].

Olive oil production in Croatia is low compared to the main olive-producing countries; however, there has been continuous growth over the last twenty years. In addition, great efforts have been made to increase oil quality, especially in Istria and the northern Adriatic islands (e.g., the islands of Krk and Cres). Historically, Istrian and Liburnian olive oils were highly valued, even at the time of the Roman Empire [2]. A long olive-growing tradition, the favourable geographic position and the climate are favourable preconditions for the production of high-quality olive oil.

Even though Italian varieties (Leccino, Pendolino, Frantolio, etc.) mostly predominate in new plantations in Croatia, cultivation of old local varieties in newly planted orchards has recently increased due to oil quality and consumer demand for typical olive oils from a specific origin. However, except for the morphological data of domestic cultivars, there is still limited data on their biological variability, origin, selection and molecular variability. The genotyping of Croatian olives by microsatellite markers has been carried out thus far on a smaller number of cultivars [3,4,5,6,7]. In Istria, Buza is the major autochthone cultivar growing only in this zone, while Drobnica is cultivated over all Croatian coastal zones. There is no official documentation of the coverage with these cultivars within their native growing areas. 

The fruit ripeness of both cultivars is considered as “middle late”, for mixed uses (as table olives and olives for oil extraction). The oils are of high oxidative stability, and their organoleptic characteristics are very appreciated by consumers [8,9,10]. Drobnica gives an oil of pronounced bitterness, gentle bitterness intensity, and a somewhat pronounced sweetness. On the other hand, Buza gives an excellent oil that depends upon the harvesting time: early harvest gives an oil of extraordinary quality, a fresh smell, and a pleasant and a pronounced bitterness; whereas a later harvest gives a sweet, rounded, oil with a fruity aroma [11].

Metabolic processes during fruit development and ripening results in changes in the size and colour of fruits, texture, composition, flavour and accumulation of oil in the mesocarp. The composition of olive oil depends on those processes. The maturation period of olive fruits lasts up to 180 days after flowering (DAF), and their development varies to the growing area, olive variety, climatic conditions and cultural practices [12]. During ripening, important chemical changes occur inside the drupe that relates to the synthesis of organic substances, especially triacylglycerols, as well as the different enzymatic activities [1] that may affect olive oil quality [13]. In the northern Adriatic region, olive harvesting begins at the end of November when the fruit is ripe or dark coloured, however, this rule has been altered in the last 20 years. More recently, the time of olive harvesting has shifted to early in October when the fruit is green or spotted. Despite existing reports about the ripening of local olive cultivars [9,11,14,15], and the chemical and sensory characteristics of Croatian olive oils [8,16,17,18,19,20,21,22], these monovarietal oils still are not sufficient investigated.

The aim of this work was to provide a report about the chemical characterisation of oils obtained during the maturation period for two Croatian cultivars, Buza and Drobnica, for one crop season. This data will be useful in profiling the typical monovarietal olive oils from these two Croatian cultivars from the northern Adriatic region.

## 2. Materials and Methods

### 2.1. Plant Materials, Sampling and Harvesting

**Figure 1:**
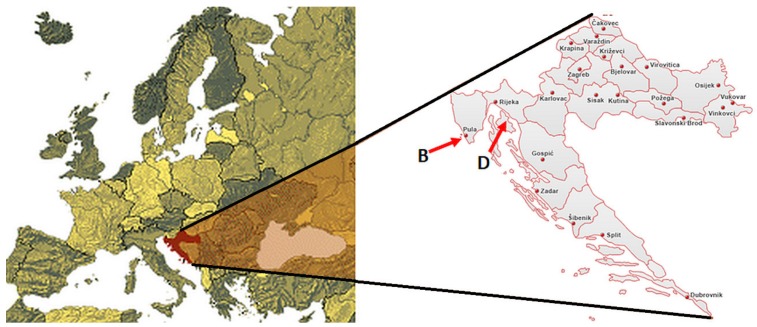
Geographic area of cultivation of Drobnica (D) and Buza (B) cultivars.

Two Croatian cultivars, Buza (or Buža) and Drobnica were chosen in this study during the crop season 1998/1999. Trees of individual variety were non-irrigated and located in orchards in their characteristic production zone (see Figure 1) in the Croatian northern Adriatic region: Buza in Vodnjan (Istria, 44°57′40″ N, 13°51′10″ E) and Drobnica in Punat (Island Krk, 45°0′36″ N, 14°37′48″ E). The plots of each variety studied were characterised by similar pedoclimatic conditions.

Fruits were randomly handpicked from all sides of a single tree of each variety, ranging from 500 to 700 g of olive fruits in order to be a representative sample during the ripening period from September to November. Fertilisers and pesticide treatment was excluded during the study crop season. Only healthy fruits, without any kind of infection or physical damage, were processed. Olive classification based on the skin colour ranged from green to completely dark purple according to Garcia et al. [23].

The collected samples from each variety were placed in polyethylene bags and maintained at +4 °C in a dark place until the next day when oil was extracted.

### 2.2. Chemicals and Standards

All standards of fatty acid methyl esters were purchased from Sigma (St. Louis, MO, USA). Methanol, *n*-hexane, petroleum ether, chloroform, potassium iodide, Folin–Ciocalteu reagent, sodium carbonate, starch, concentrated sulphuric, nitric and hydrochloric acid (37%) were purchased from Kemika (Zagreb, Croatia). Anhydrous sodium sulphate and 2,2-diphenyl-1-picryl-hydrazyl (DPPH) were purchased from Sigma (St. Louis, MO, USA). 

Sodium and potassium hydroxides in the form of pellets, chloroform and analytical standards (dihydrocholesterol, campesterol, stigmasterol, β-sitosterol, eicosanol, docosanol, tricosanol, tetracosanol, pentacosanol, hexacosanol, heptacosanol, octacosanol, squalene and (±)-α-tocopherol) were purchased from Sigma (St. Louis, MO, USA). Ethanol and a silylating mixture according to Sweeley were purchased from Fluka (Buchs, Switzerland).

Hydroxytyrosol, tyrosol, oleuropein, lutein and apigenin were purchased from Extrasynthese (Genay, France). Caffeic acid and gallic acid were purchased from Sigma (St. Louis, MO, USA). Standard stock solutions of each compound were 1 mg/mL in methanol. 

All mineral standards were purchased from Merck (Darmstadt, Germany) and diluted to the appropriate obtained concentration.

All standards stored at +4 °C in amber vials and in a dark place until analysis. 

### 2.3. Sample Preparation and Chemical Analysis

The olives of each cultivar were blended in a laboratory blender (Vitamix 5000, Vitamix, Waterloo, ON, Canada) to obtain a homogeneous olive-paste, after which the olive oil was extracted from olive-paste with *n*-hexane, filtered throughout sodium sulphate and, in order to obtain the olive oil, *n*-hexane is removed using a rotary evaporator to a given constant oil weight. Olive oil was stored in a dark glass bottle and left in a dry atmosphere until the analyses were performed.

#### 2.3.1. Analytical Indices

General indices of quality in virgin olive oil (degree of acidity, peroxides value) was determined according to the methodology described in Regulation (EEC) 2568/91 [24]. 

Free acidity (FA) and peroxide value (PV) were immediately determined while other parameters were determined a week after the oils were extracted. All parameters were determined in triplicate.

#### 2.3.2. Fatty Acid Analysis

In brief, fatty acid composition in extracted olive oils was determined using a gas chromatograph Autosystem XL (Perkin–Elmer, Norwalk, CT, USA) with a flame-ionisation detector (FID) according to the EC Regulation 2568/91. The chromatography software Perkin–Elmer Nelson (Turbochrom 4, rev.4.1.) was used for data acquisition and processing. Hydrogen was obtained by using a Claind hydrogen generator (Claind Srl, Tremezzina, Italy). 

Standard of each fatty acid in the form of fatty acid methyl esters (FAME) was analysed using a capillary column SP-2330 (Supelco, Bellafonte, PA, USA), 30 m × 0.32 mm i.d., of 0.2 µm film thickness. Helium was used as the carrier gas with split injection (100:1). The analyses were carried out in the programmed temperature mode from 140 °C to 220 °C, with a rate of 5 °C/min and then in the isothermal phase for 25 min. The detector temperature was 350 °C and the injector temperature was 300 °C. The same method was used in the analysis of the composition of fatty acids in olive oil. The results were expressed as a percentage of individual fatty acids (myristic, myristoleic, palmitic, palmitooleic, stearic, oleic, linoleic, arachidic, linolenic, eicosenoic, behenic and lignoceric).

#### 2.3.3. Analysis of Unsaponifiables

The alkanols, squalene, α-tocopherol and sterols in extracting olive oils were determined by direct method involving gas chromatographic (GC) analysis of the unsaponifiable fraction after silylation [25]. 

A capillary column SPB-5 (Supelco, Inc., Bellafonte, PA, USA), 30 m long, 0.53 mm i.d., 0.5 µm film thickness was used. The gas chromatographic conditions were as follows: oven temperature programmed from 180 °C to 270 °C with a rate of 8 °C/min and then in the isothermal phase for 65 min. The detector temperature was 300 °C and the injector temperature was 290 °C. Total sterols, total alkanols, α-tocopherol and squalene were expressed as mg/kg of olive oil. In addition, individual sterols and alkanols were expressed as a percentage concentration (%) in the total sterols and total alkanols. α-tocopherol and squalene were determined simultaneously in the same fraction and analysis.

#### 2.3.4. Analysis of Phenols in Olive Oil Extracts

Briefly, 4.5 g of olive oil samples were dissolved in 15 mL 80% aqueous methanol and shaken for 1 min. The mixtures were placed in an ultrasonic bath at room temperature for 20 min and centrifuged (at 5000 rpm) for 25 min. Removed supernatant was filtered through a 0.45 mm nylon filter membrane and washed with *n*-hexane (three times). The collected methanolic extracts were evaporated to a given dryness in a rotary evaporator. Dry residues were reconstituted with an equal volume of HPLC-grade methanol for total phenolic (TP) determination, DPPH scavenger activity and HPLC analysis.

##### Total Phenols

The microplate method was used for the determination of total phenolic (TP) content. In each well was added 20 µL of the standard sample and 100 µL of diluted Folin–Ciocalteu reagent (1:10). The mixtures were quickly shaken, and put in the dark for 5 min, after which was added 80 µL 7.5% (*w*/*v*) sodium carbonate and a microplate covered left in the dark at room temperature for 2 h. 

Absorbances were read using a 750 nm filter at ELx808 Absorbance Reader (BioTek Instruments, Inc., Winooski, VT, USA). Gallic acid was used as the standard in a concentration range of 7.8–2000 µg/mL and total phenols were expressed as the gallic acid equivalent (GAE) per kg of olive oil. Each standard concentration and samples were determined in duplicate.

##### DPPH Scavenger Activity

The microplate method was used for the determination of the antioxidative activity in olive oils by means of the DPPH method. In each well was added 22 µL of standard sample and 200 µL of 0.1 mM DPPH reagent. After shaken, the mixture in the microplate was covered and incubated in a dark place at 37 °C for 30 min. Absorbances were read using a 515 nm filter of ELx808 Absorbance Reader (BioTek Instruments, Inc., Winooski, VT, USA). DPPH scavenger activity was expressed as a percentage of the scavenger activity related to the DPPH reagent. All samples were determined in duplicate.

##### HPLC Analysis of Phenols

A TSP Spectra System (Thermo Separation Products Inc., San Jose, CA, USA) with P2000 binary pump, SCM1000 vacuum membrane degasser, Rheodyne injector 7725i, equipped with ChromQuest software and a UV2000 detector with a SN4000 controller was used for the analysis of olive oil phenolic extracts. Separation was achieved on a Supelco C18 HPLC column (4.6 mm × 250 mm, 7 µm particle size), operated at 25 °C. The mobile phase was 2% acetic acid in water (solvent A) and methanol (solvent B) at a flow rate of 1 mL/min. The gradient started with 0% B to reach 5% B at 3 min; 20% B at 18 min; isocratic for 2 min; 40% B at 30 min; 50% B at 40 min; 100% B at 50 min and isocratic for 10 min. A total run was 60 min. Chromatograms were recorded at 280 nm [26].

Individual stock standard solutions, as well as multi standard solutions, were prepared by dilution of each compound in methanol. Standard solutions were stored at −20 °C.

Dry matters (DM) of two replicate olive oil extracts were dissolved in methanol and 20 µL of was injected. The identification of the separated compounds was carried out by retention time mapping with a set of standards dissolved in methanol. Quantification was performed by calibration curves using caffeic acid at 280 nm as an external standard (ranging from 5–1000 µg/mL, R^2^ = 0.9976). All data presented are mean values of two independent experiments.

#### 2.3.5. Analysis of Minerals

The dry ashing procedure was applied to determine the minerals. A sample of 10 g of olive oil aliquot was exactly weighed in a 50 mL porcelain crucible. The crucible was transferred to a heating plate and the temperature slowly increased until the sample was completely carbonised. The carbonised material was then burned in a muffle oven by slowly increasing the temperature up to 500 °C, which was maintained until white ashes were obtained. The crucible was cooled to room temperature and ashes were dissolved in 10 mL hot 50% (*v*/*v*) hydrochloric acid that was quantitatively transferred to a 10 mL volumetric flask. The volume was filled up to the mark with hot 50% (*v*/*v*) hydrochloric acid. Liquid samples were introduced into the ICPS apparatus by pneumatic nebulisation. Measurements were performed in a Philips PU 7000 ICP Spectrometer (Unicam Analytical Systems, Cambridge, UK) via ASTM D 19756-91 method (1 kW power, 12 L/min coolant, 38 psi nebuliser pressure). Standard solutions for each mineral (Na, K, Ca, Mg, Fe, Zn, Cu, Pb, Cd, Ni, Cr, Ba, Al and P) were prepared for the calibration curves under the same conditions. The glassware was washed with detergent, soaked in 15% (*v*/*v*) nitric acid 24 h, rinsed with deionised distilled water and dried before use for mineral analysis.

### 2.4. Statistical Analysis

The data were evaluated with Statistica (data analysis software system), version 13 (TIBCO Software Inc., 2017, Palo Alto, CA, USA). The statistical analysis was performed using the nonparametric Kruskal–Wallis Anova by Ranks and Kruskal–Wallis Multiple Comparisons *P* values (2-tailed) between ripening stages to assess significant differences among the picking date and individual chemical parameters determined in the obtained oils. The Mann–Whitney U-test was performed for estimation of significant changes between Buza and Drobnica obtained oils at the same ripening stage. Statistical significance was assumed, given *P* < 0.05, and the data are reported on the basis of mean (SD).

## 3. Results and Discussion

### 3.1. Fatty Acids

Optimal harvesting time is important due to the tendency for the production of high yield oil and high-quality extra virgin olive oil. But, these requirements are not easy to meet. 

Presented results followed the previous research of the physico-chemical characteristics of oil extracted from the pulp of the olive Buza during ripening in the year 1998 in the same orchard [9]. The climatic conditions during olive harvesting in the crop years 1998 and 1999 were different, and therefore also the changed acidity and peroxide value (see Table 1). In the year 1999, a higher average temperature and significantly lower rainfall than in the year 1998 were noted, when a higher polyunsaturated fatty acids (PUFAs) in all olive picking dates were reported. However, the level of monounsaturated fatty acids (MUFAs) and oleic acid in olive oil did not change significantly during ripening (see Table 2), which is in accordance with earlier results [9]. 

We found that olive oil from Buza has a significantly higher level of oleic acid than Drobnica, which corresponds with results from the multi-year data ranging from 1992 to 2009 in Istria where the level of oleic acid in the Buza variety was 74% [20] or near 74% [11]. In Drobnica, cultivated in Dalmatia, oleic acid was found to be lower (near or under 70%) [7,8].

Changes in the fatty acid composition of the extracted oil have been reported to be associated with olive fruit maturation [27,28]. Stearic acid (C18:0) was minimally changed in Drobnica (from 2.39 ± 0.10 to 1.98 ± 0.26), while in Buza it was reduced (from 2.12 ± 0.16 to 1.67 ± 0.39) with maturation. The ripening process changed significantly the myristoleic, linoleic, linolenic, eicosenoic and lignoceric acids in oil from Buza, and oleic and linolenic acid in oil from Drobnica. More differences between Buza and Drobnica oils were found in November, as a result of its variability and also due to differences in the ripening process. Palmitic acid content decreased during ripening in Buza oils, from 13.44 ± 0.25 to 12.07 ± 1.58, while it increased in Drobnica oils: from 15.11 ± 1.17 to 18.48 ± 3.37. In addition, linoleic acid and oleic acid showed the opposite trend: almost unchanged linoleic acid in Drobnica oil and a significant increase of the same in Buza in November. The same trend was found in PUFAs.

The ratio of MUFAs/PUFAs was attributed the nutritional properties and oxidative stability of olive oils. This ratio was higher in Buza in September-October (9.26 ± 0.07 to 9.23 ± 1.20), however, in November it notably dropped down (to 6.85 ± 0.72) under the value of Drobnica (7.38 ± 0.58). An increase in PUFAs negatively affects the olive oil stability observed in November in Buza. On the other hand, this trend is not attributed to Drobnica. 

Ipek et al. [29] reported the association between fatty acid traits and simple sequence repeat (SSR) markers. Significant associations were found between five SSR markers and the stearic, oleic, linoleic, and linolenic acids of olive oil. Also, very high associations (*P* < 0.001) were indicated between ssrOeUA-DCA14 and stearic acid; and between GAPU71B and oleic acid, so that these markers could be used for marker-assisted selection of olives.

### 3.2. Unsaponifiable Compounds

Phytosterols, alkanols, squalene, tocopherols are the main groups of components analysed in the unsaponifiable fraction. The sterols and alcohols profile is used for characterisation of virgin olive oils and detection of the adulteration of olive oil with similar vegetable oils or virgin olive oil with olive-pomace oil [30,31].

The amount of unsaponifiable matter in olive oil varies from 1% to 5% in ripe olives [32]. The higher content of unsaponifiables is probably associated with olive processing as well as the subsequent loss of their physiological activities and quality. As shown in Table 3, the level of unsaponifiable matter in laboratory extracted oil in Drobnica ranged from 1.90 ± 0.09% to 2.58 ± 0.11%, and from 1.47 ± 0.08% to 2.37 ± 0.11% in Buza. Unsaponifiable components can provide information about the adulteration of vegetable oils as well as their variety and even the geographical origin, as has been analysed in some publications [33,34,35,36,37,38]. 

Total sterols significantly changed during ripening in both cultivars studied. Their content decreased from September to November in Drobnica from 2388.36 ± 444.42 mg/kg to 968.68 ± 31.71 mg/kg, and in Buza from 1354.47 ± 90.58 mg/kg to 850.19 ± 94.23 mg/kg. Although the level of total sterols varied between cultivars, only β-sitosterol in Buza changed significantly during ripening. As a predominant phytosterol in both oils, β-sitosterol ranged from 95.24 ± 2.73% to 98.27 ± 1.18% in Drobnica and from 93.95 ± 0.30% to 98.09 ± 0.25% in Buza. Our results of the total sterols, β-sitosterol and squalene showed the opposite trend than that described by Fernández-Cuesta et al. [39]. Squalene is the major olive oil hydrocarbon accounting for more than 90% of the hydrocarbon fraction [33]. In the oils from Buza and Drobnica, notable differences were found in the squalene content as shown in Table 3. Squalene was increased in Drobnica from September (6927.46 ± 1878.75 mg/kg) to October (9696.52 ± 299.22 mg/kg), after which it decreased in November (5078 ± 1598.01 mg/kg). Buza showed a rise in squalene content during all periods observed (5383.73 ± 576.25 mg/kg–7696.5 ± 503.15 mg/kg). Fernández-Cuesta et al. [39] reported that the level of squalene increased during the period of maturation (September–November) in the cultivars Picual and Arbequina grown in Cordoba (Spain), which significantly increased from September (4102 mg/kg) to November (4673 mg/kg). However, they found no difference in the fruit flesh between November and December. Squalene also had no effect on the oil oxidative stability in the case of Drobnica and Lastovka [7]. Beltrán et al. [40] studied 28 olive cultivars from the World Olive Germplasm Collection of Instituto de Investigación y Formación Agraria, Pesquera (IFAPA) in Cordoba, where from 110 to 839 mg/100 g of squalene was found in virgin olive oils. The difference in squalene content was explained by the genetic variability. It is worth noticing that the sterol content of the oil varies even within the fruits or nuts collected from the same tree [41].

Tocopherols and polar phenolic compounds are responsible for the oxidative stability of the olive oil. In general, tocopherols decreased during the ripening process [42,43]. We found that the concentration of α-tocopherol was altered significantly only in Buza. A decrease of α-tocopherol was also observed in both cultivars during ripening (in Buza it ranged from 141.74 ± 7.70 to 64.97 ± 15.42 mg/kg, and in Drobnica from 154.64 ± 54.66 to 77.55 ± 5.60 mg/kg). The concentration of α-tocopherol in virgin olive oils depends on many factors (cultivars, geographic area, oil processing, irrigation, etc.) including genetic factors [43,44]. The level of rainfall has an effect on α-tocopherol content, thus, the drier crop years indicated a greater tocopherol concentration. However, this effect was dependent upon the cultivar [43].

Despite the lower contribution of α-tocopherol in comparison to phenolic compounds in maintaining oxidative stability, α-tocopherol is still important, especially as oils ageing progresses [44]. 

n-alkanes from nC22 to nC33 is characteristic for olive oil which makes it different from other vegetable oils. The profiles of aliphatic alcohols in olive oil also depend on the origin and fruit variety, so it is linked to the authenticity of the extra virgin olive oil. The Greek extra virgin olive oils were typically characterised by high levels of nC23 and nC25. However, the carbon number profile of Italian and Spanish oils was not characterised by a single profile [34]. 

The level of total alkanols was significantly different in Buza during ripening. As shown in Table 3, in both cultivars significant differences in nC23, nC24, nC26 and nC28 were found in all examined ripening stages, while nC22 was changed significantly only in Buza. Hexacosanol and tetracosanol were predominant aliphatic alcohols in both cultivars: hexacosanol was the highest in September (67.97 ± 1.44% in Drobnica and 63.14 ± 5.77% in Buza) and October (48.49 ± 1.10% in Drobnica and 64.18 ± 2.25% in Buza) and tetracosanol was the highest in November (48.25 ± 4.34% in Drobnica and 41.42 ± 0.40% in Buza). It is observed that Drobnica has an adequate trend of declining hexacosanol with ripening different from Buza. In addition, Drobnica had a higher amount of total alkanols in relation to Buza. 

Koprivnjak et al. [18] have used fatty acids and hydrocarbons to compare the Croatian varieties Bianchera, Carbonazza and Busa with the Italian Leccino variety. They have concluded that low values of aliphatic hydrocarbons nC24 characterise autochthonous Croatian varieties of cultivar, while higher values of nC25 and nC35 hydrocarbons characterise the Italian Leccino. We found lower values of nC24 at early stages of olive ripening. Our results also suggested that changes in aliphatic alcohols are bigger in Buza than in the Drobnica cultivar during ripening.

Based on these results, it is also concluded that sterols and alcohols rather than fatty acids are both very important compounds for the chemical authentication of virgin olive oil varieties [45].

In many cultivars, hexacosanol was found as the most predominant of all fatty alcohols. Giuffrè [46] found hexacosanol to be the major alkanol in olive oil in three autochthonous cultivars (Cassanese, Ottobratica, Sinopolese) and seven allochthonous cultivars (Coratina, Itrana, Leccino, Nocellara Messinese, Nociara, Pendolino and Picholine) with regard to olive oils from Southwest Calabria. Next, hexacosanol has found to be the most common aliphatic alcohol in monovarietal virgin olive oil from Tunisian cultivars (Jdallou, Chemlali Sfax, Swabâa, El Hor, and Oueslati) [47] in the olive oil of cultivars grown in Central Italy, including the Leccino cultivar [48]; in Coratina from the Apulia Region in the Southeast of Italy; in Koroneki from Crete [49]; in Arbequina, Picual, and Manzanilla [50]; and in pomace olive oil [51]. 

López-López et al. [32] observed the significant effects of processing on unsaponifiable matter (β-sitosterol, Δ5-avenasterol, total sterols, docosanol, tetracosanol) in the Manzanilla and Hojiblanca cultivars. In addition, Ranalli et al. [52] reported on the greater concentration of fatty alcohols in oil from cultivars grown in central Italy (500 mg/kg) as compared with those grown in southwest Calabria. On the other hand, Apparicio and Luna [49] noted that in the olive oil of Coratina grown in Apulia, the level of total aliphatic alcohols, 63 mg/kg (in two-phase extraction) and 58 mg/kg (in three phase extraction), is less than those grown in southwest Calabria. Our results have shown that the highest amount of aliphatic alcohols in oil is obtained from Drobnica (236.06 mg/kg), whereas it was determined to be the lowest in October in Buza (42.81 mg/kg). Angerosa et al. [53] studied the influence of rainfall on the synthesis of oil on the Italian cultivar Frantoio of varying geographic origin. By applying statistical procedures, the authors have found that the amounts of sterols, squalene, oleic acid and some triacylglycerols were explained by the autumn temperatures, the relative humidity of the summer months and the rainfall over the whole year.

### 3.3. Phenolic Compounds

Phenolic compounds, α-tocopherol and β-carotene are reported as the main groups of compounds with antioxidant properties that correlated to the oxidative stability of virgin olive oils [54,55].

The results presented in Table 4 show a significant change in total phenols and their antioxidant activity during ripening in both cultivars. Drobnica had a higher content of total phenols during the examined ripening period (437.67 ± 10.50 mg/kg, 316.34 ± 4.70 mg/kg and 273.26 ± 7.14 mg/kg, respectively) as compared with Buza (374.98 ± 10.49 mg/kg, 289.96 ± 9.79 mg/kg and 250.09 ± 5.52 mg/kg, respectively). A high level of phenolics is also connected with the high antioxidant activity of Drobnica, which ranged from 71.18 ± 0.98% to 57.31 ± 0.96% and in Buza from 71.48 ± 1.16% to 61.14 ± 0.48%. The concentration of hydroxytyrosol (HYTY) and tyrosol (TY) was significantly changed in both cultivars during ripening, while the concentration of luteolin (Lut) and apigenin (Apig) was significantly altered only in oil from Buza. The higher level of HYTY was found in Buza in September, while in November it was determined in Drobnica. The level of oleuropein (OL) was higher in Drobnica in September and October, however, in November it was almost the same in both cultivars. 

Bilušić et al. [7] reported that monovarietal extra virgin olive oil (EVOO) from Drobnica contained the highest amount of total phenols and major secoiridoid derivatives (oleocanthal, oleacein, oleuropein aglycon, and ligstroside aglycon) compared to other studied monovarietal EVOOs. This also affected the highest antioxidant activity of Drobnica oil and it’s very long (23 h) oxidative stability as determined by the Rancimat method. Similarly, Dalmatian monovarietal olive oils were analysed in relation to the harvest period (Buhavica, Drobnica, Lastovka and Oblica) [8]. It was observed that the late harvest contained an extremely high concentration of oleocanthal + oleacein (966 mg/kg) in EVOO from Drobnica, and that oils from Drobnica and Lastovka (Korčula Island, South Dalmatia, position 42°57′31.62′′ N) had the longest oxidative stability (20.95 and 18.65 h, respectively). This study showed that the level of phenolics depends on the cultivar, however, the authors did not determine a significant change of phenolic secoiridoids in oil from Drobnica during the harvest period. 

Lukić et al. [56] studied the concentrations of phenols and volatiles in virgin olive oil from a late-ripening olive cultivar Istarska bjelica from October to December in the crop season 2015 grown in an unirrigated olive orchard located at the position 45°13′32.99′′ N, 13°35′38′′ E (Poreč, Croatia). They found that TY, HYTY increased during ripening, while other important phenolic compounds were decreased such as a dialdehydic form of decarboxymethyl elenolic acid linked to hydroxytyrosol (3,4-DHPEA-EDA or oleacein), a dialdehydic form of decarboxymethyl elenolic acid linked to tyrosol (p-HPEA-EDA or oleocanthal), and oleuropein and ligstroside aglycones. Total secoiridoids and total phenols also were decreased during ripening. In addition, the authors found significant changes in the interaction between malaxation temperature, malaxation time and ripening both in relation to simple and complex phenols. Similarly, these authors studied the Dalmatian cultivar Oblica from September to November 2015 grown in Kaštel Stari at the position 43°32′59.99′′ N, 16°20′59.99′′ E [14]. The above-mentioned results have confirmed the existence of a geographic and genotype difference between these two Croatian cultivars. This was especially observed in the opposite trend in p-HPEA-EDA, which is increased during ripening in Oblica. Malaxation temperature, malaxation time and ripening also affected phenolics composition in the oils.

According to the results based on the ripening process from November to January, Bengana et al. [57] suggested that a maturation index of 2.4, found in November, was the most appropriate for the harvesting of olives in order to obtain the high-quality EVOOs from the Chemlal cultivar grown in an orchard located in the Haizar area in north-central Algeria. The highest HYTY, TY, phenolic alcohols and secoiridoids were noted in November. However, significant variation in olive-oil yield, carotenoids, and tocopherol content was not observed.

The secoiridoid derivatives of HYTY and TY were the major phenolic compounds in oils from Ayvalık, Domat and Gemlik olive varieties collected at different ripening periods from August to December in Edremit (Balıkesir) in Turkey in 2006 [58]. In all examined oils the greatest concentration of HYTY was found in October (0.80 mg/kg, 1.15 mg/kg, 0.63 mg/kg, respectively). The level of luteolin in oils increased with the ripening of Ayvalık, Domat and Gemlik olives, ranging between 0.27–2.28, 0.00–1.42, 0.28–1.74 mg/kg, respectively. On the other hand, the highest TY content was established in Gemlik oil in August. The level and profile of other determined phenolic compounds depended on varietal differences during ripening.

Gomez-Rico et al. [59] studied the degree of ripening of the olive fruit on the biophenolic and volatile profiles of six different Spanish varieties (Arbequina, Cornicabra, Morisca, Picolimón, Picudo and Picual) and their corresponding virgin olive oils. They found that the ratio between biophenol content in the olive fruit and its resulting olive oil varied significantly for each of the cultivars studied, especially in Picudo and Picolimón (ranging from 2.3 to 28, respectively). Besides the statistical difference in oleuropein content in all varieties studied, demethyloleuropein was only found in the Arbequina variety during the ripening process. They also established a different concentration of HYTY and TY dependent upon variety: 2.9 and 2.1 mg/100 g HYTY in Arbequina, 2.8 and 2.1 mg/100 g in Cornicabra, 0.4 and 0.6 mg/100 g in Marisca, 0.8 and 0.6 mg/100 g in Picolimon, 1.8 and 2.2 mg/100 g in Picual olive varieties, respectively; and TY content 2.4 and 2.1 mg/100 g in Arbequina, 1.5 and 1.2 mg/100 g in Cornicabra, 5.5 and 6.4 mg/100 g in Morisca, 4.2 and 3.9 mg/100 g in Picolimon and 3.3 and 3.3 mg/100 g in unripe and ripe Picual olive varieties, respectively.

High content of lipophilic (>300 mg/kg) and hydrophilic phenols (>600 mg/kg) for Galega Vulgar and Cobrançosa olive oils corresponded with early ripening stages were found Peres et al. [60]. Total phenols were decreased when the ripening index ranged from 2.5 to 3.5. The dialdehydic form of elenolic acid linked to hydroxytyrosol (3,4-DHPEA-EDA or oleacen) was the major phenolic compound identified in both oils, and the concentration of hydroxytyrosol and tyrosol was very low due to the high levels of 3,4-DHPEA-EDA and p- HPEA-EDA as their esterified derivatives.

The mentioned results confirm that changes in fruit colour during development and ripening olive fruits are crucial to the antioxidant capacity of oils. During harvesting, the content of TY and HYTY undergoes change. In early harvest, they were higher, and in late harvesting, HYTY levels decreased by 50.40% [61]. HYTY is one of the phenols with the highest antioxidant effect in olive oil [62], which results in the higher oxidative stability of the oil. This is in accord with the results obtained by Martinez Nieto et al. [63], who reported that tyrosol and hydroxytyrosol concentrations decreased with increasing olive ripeness in the Picual and Arbequina varieties.

A high correlation between the antioxidant capacity of the chloroplastic pigments and total phenolic compounds in the Arbequina variety reported by Fernandez-Orozco et al. [64]. The antioxidant capacity was increased with a higher total chlorophyll and xanthophyll content, while low correlation was found with β-carotene content. That confirms the fact that the early ripening stage is rich in antioxidants.

### 3.4. Minerals

All plants must obtain a number of macro and microelements from their environment to ensure successful growth and development of both vegetative and reproductive tissues. Nitrogen, sulphur, and phosphorus serve as constituents of proteins and nucleic acids, while magnesium and the micronutrients (except chlorine) may function as constituents of organic structures, predominantly of enzyme molecules, where they are involved in the catalytic functions of the enzymes [65].

Our hypothesis on the different content of minerals in olive oils during ripening was based on (i) variable soil mineral content in orchards, and (ii) different physiological mineral requirements in fruit during the ripening process. We assumed that the mineral profile in the oils correlates with the mineral content in the olive fruit. However, in the literature we did not find any report about the variation of mineral content in oils obtained from olive fruit during ripening. To be able to compare the mineral levels in oils, it is important to emphasise that the olives were not treated with fertilisers or metal-containing pesticides, and wherein the method of oil preparation could not influence the increase of trace elements. 

In some cases, traces of Fe and Cu in virgin olive oil may originate from the soil and fertilisers, agrochemicals or from contamination by processing equipment and storage containers. 

Table 5 presents the trace elements in the olive oil extracted from Drobnica and Busa olives during ripening. In both orchards, the soil is rich in Ca and Fe. The orchard where Drobnica grew was near the sea, which reflected in a greater Na concentration. On the other hand, the level of Ca was higher in Buza that was located six kilometres from the sea. During the ripening, significant differences in both varieties were found in the content of Na, Mg, Fe, Zn, Cu, Al and P, as well as K in Drobnica and Ca in Busa. In both cultivars were found the lowest values of Na, K, Mg, Cu, Al and P in October, after that, in November the level of Ca, Zn, Cu and Ni was increased in Drobnica and the level of Fe was decreased in Buza.

Maintenance of minerals by means of mineral nutrition is a prerequisite for providing co-factors for the many enzymes of the phenylpropanoid and flavonoid pathway. Mg and Mn ions ensure the functioning of phenylalanine ammonia lyase (PAL), of CoA-ligases, and of methyltransferases [66]. This would mean that the Mg level can affect the level of phenolic compounds, especially in the early stage of ripening as presented in Table 4 and Table 5. Three forms of the superoxide dismutase (SOD) exist in plants as enzyme antioxidants, classified by their active site of the metal ion as Cu/Zn, Mn, and Fe forms. Thus, the need for these metals exists during ripening. Besides its inclusion in the Mn-containing superoxide dismutase (SOD), Mn plays an important role in the water-splitting enzyme of photosynthesis associated with photosystem II [67]. 

The water splitting site consists of a cluster of four Mn ions and a Ca ion surrounded by amino acid side chains, of which seven provide ligands to the metals [68]. Photosynthesis is the most sensitive process for manganese deficiency in higher plants. In Mn deficient leaves not only the chlorophyll content is lower, but also the content of typical thylakolid membrane constituents (glycolipids and polyunsaturated fatty acids) can be depressed up to 50%, which can be attributed to the role of Mn in the biosynthesis of fatty acids and carotenoids and related compounds [65].

Although we did not find data reports about the level of minerals during ripening in the olive fruit, there are such reports that are related to other fruits.

The effect of increasing avocado fruit maturity on mineral composition and phenolic content resulted in an influence on postharvest fruit quality and the ripening physiology of fruit. Reduced calcium and magnesium concentrations were found in fruits with increased maturity [69]. An increase in the concentrations of calcium, magnesium and phosphorus with the ripening process of the asparagus was found. The green ripening state has a greater concentration of calcium, magnesium and phosphorus. The changes from the white asparagus into a green ripening state affect a decrease in the content of sodium, while no significant differences were established for potassium [70]. 

Much evidence was also given as to the level of the trace elements in some commercial olive oils in relation to olive oil processing, geographic origin, harvest year and olive cultivars [71]. In a total of 50 samples of monovarietal EVVO analysed from two Protected Designation of Origin (PDO) Spanish provinces, Granada and Jaén, there were found to be significant differences between Cu, Cr, Fe and Ni content according to the geographical origin of the oils but not for Mn content [72]. The authors suggest that the trace element content of extra virgin olive oils based on their geographical origin can be used for their local characterisation.

## 4. Conclusions

The prediction of optimum harvest timing is a key factor of the balance between oil quality and quantity. Many factors influence the quality of olive oil including: the cultivar, the geographic plantation area, the olive oil processing method, and the climate and harvesting conditions. Therefore, the present paper contains new information about the major and minor compounds of oil obtained from the two Croatian cultivars, Drobnica and Buza, during the olive fruit ripening process. Although the data in this study related to the crop year 1998/1999, they are unpublished and valuable in increasing the knowledge of the determination of variability in these cultivars.

The higher oleic acid was observed in oil from the Buza cultivar (ranging from 73.91 to 74.86%). The content of oleic in Buza and the content of linoleic acid in Drobnica did not change significantly during ripening period. Nevertheless, this study evidenced that the oleic/linoleic acid ratio decreased as the olives ripened, especially in Buza where this ratio was significantly reduced. The sterols, α-tocopherol, squalene, aliphatic alcohols and phenolic compounds of the olive oil can be used as reliable indicators of the variety and the ripening process of Buza and Drobnica. Based on these data, we determined that October was the optimal harvesting period for both cultivars and that this date also coincided with fruit skin colour (green to spotted). 

A lower concentration of squalene, total sterols and aliphatic alcohols were also found in Buza. Hexacosanol and tetracosanol were the predominant alkanols observed in the oils of both cultivars. Hexacosanol levels showed a decrease while tetracosanol levels increased during ripening.

With regard to antioxidants, Drobnica oil had the highest level of α-tocopherol and total phenols, however, Buza had a little higher DPPH activity in the ripening progress when the concentration of HYTY was the highest.

Finally, trace minerals detected in Buza and Drobnica oil differed, which can be an indicator of oxidative stability and authenticity of oils.

## Figures and Tables

**Table 1:** Climatic conditions during the harvesting of Drobnica and Buza cultivars from 1998–1999.

	1999			1998		
Months	T (°C)	Humidity (%)	Rainfall (mm)	T (°C)	Humidity (%)	Rainfall (mm)
August	25.3	49.9	2.7	26.4	39.4	45.6
September	22.1	55.9	9.5	19.0	55.4	423.9
October	16.0	61.2	16.7	15.2	60.9	283.1
November	11.5	47.7	65.2	8.7	46.1	97.6

T = means of monthly temperature; Humidity = means of monthly percentage of humidity; Rainfall = means of monthly rainfall (mm of water).

**Table 2:** Quality parameters and fatty acid composition (%) at various ripening stages of Drobnica and Buza.

	September		October		November	
	DROBNICA (20/09/1999)	BUZA (21/9/1999)	DROBNICA (09/10/1999)	BUZA (14/10/1999)	DROBNICA (3/11/1999)	BUZA (7/11/1999)
FA	0.47 ± 0.10	0.32 ± 0.01	0.19 ± 0.00	0.23 ± 0.02	0.26 ± 0.00	0.19 ± 0.01
PV	3.95 ± 0.35	0.70 ± 0.01	2.88 ± 1.44	0.68 ± 0.00	1.35 ± 0.17	0.99 ± 0.04
*Fatty acids (%)*						
Myristic (14:0)	0.03 ± 0.00	0.02 ± 0.02	0.03 ± 0.01	0.01 ± 0.01 ^#^	0.02 ± 0.00	0.00 ± 0.01 ^$^
Myristoleic (14:1) ^B^	0.01 ± 0.01	0.02 ± 0.00	0.01 ± 0.00	0.01 ± 0.00	0.01 ± 0.01	0.00 ± 0.00 ^c^
Palmitic (16:0)	15.11 ± 1.17	13.44 ± 0.25	16.98 ± 1.62	14.13 ± 1.96	18.48 ± 3.37	12.07 ± 1.58 ^$^
Palmitooleic (16:1)	1.34 ± 0.16	0.81 ± 0.28	2.01 ± 0.37	1.27 ± 0.51	2.84 ± 1.27	0.71 ± 0.62
Stearic (18:0)	2.39 ± 0.10	2.12 ± 0.16	2.24 ± 0.24	1.97 ± 0.24	1.98 ± 0.26	1.67 ± 0.39
Oleic (18:1)^D^	71.25 ± 1.79	74.86 ± 0.28	69.37 ± 1.19	73.91 ± 1.12 ^#^	66.77 ± 2.18 ^c^	74.31 ± 2.82 ^$^
Linoleic (18:2) ^B^	8.31 ± 0.59	7.38 ± 0.13	8.15 ± 0.74	7.67 ± 1.05	8.62 ± 0.68	10.85 ± 0.91 ^c,$^
Arachidic (20:0)	0.18 ± 0.14	0.25 ± 0.04	0.29 ± 0.11	0.24 ± 0.10	0.21 ± 0.09	0.11 ± 0.19
Linolenic (18:3) ^B,D^	1.17 ± 0.24	0.82 ± 0.10	0.73 ± 0.24	0.59 ± 0.04	0.87 ± 0.14 ^a^	0.19 ± 0.13 ^c,$^
Eicosenoic (20:1) ^B^	0.17 ± 0.15	0.25 ± 0.06	0.18 ± 0.16	0.18 ± 0.10	0.17 ± 0.15	0.05 ± 0.08 ^c^
Behenic (22:0)	0.03 ± 0.02	0.02 ± 0.02	0.01 ± 0.01	0.01 ± 0.01	0.02 ± 0.02	0.04 ± 0.06
Lignoceric (24:0) ^B^	0.01 ± 0.01	0.01 ± 0.00	0.01 ± 0.08	0.01 ± 0.00	0.01 ± 0.00	0.00 ± 0.00 ^$^
Σ SFA	17.74 ± 1.44	15.86 ± 0.04	19.56 ± 2.00	16.36 ± 1.79	20.72 ± 3.75	13.90 ± 1.97 ^$^
Σ MUFA	72.77 ± 2.10	75.94 ± 0.04	71.55 ± 1.72	75.38 ± 0.72 ^#^	69.78 ± 3.59	75.07 ± 2.21 ^$^
Σ PUFA ^B^	9.48 ± 0.83	8.20 ± 0.05	8.89 ± 0.98	8.26 ± 1.07	9.49 ± 0.82	11.03 ± 1.03 ^c^
MUFA/SFA	4.12 ± 0.36	4.79 ± 0.01	3.68 ± 0.34	4.64 ± 0.01 ^#^	3.45 ± 0.70	5.50 ± 1.00 ^$^
MUFA/PUFA ^B^	7.73 ± 0.84	9.26 ± 0.07	8.11 ± 0.85	9.23 ± 1.20	7.38 ± 0.58	6.85 ± 0.72 ^c^
16:0/18:0 ^D^	6.32 ± 0.65	6.36 ± 0.58	7.65 ± 1.30	7.31 ± 1.68	9.30 ± 0.63 ^c^	7.37 ± 1.32
18:1/18:0	29.79 ± 1.28	35.37 ± 2.77	31.17 ± 1.28	37.94 ± 4.12	34.17 ± 5.25	46.04 ± 10.75

Note: Data are expressed as mean ± SD (*N* = 3). Different superscripts mark significant difference (*P* < 0.05), as follows: ^B^ determined by Kruskal–Wallis Anova by Ranks for Buza; ^D^ determined by Kruskal–Wallis Anova by Ranks for Drobnica; ^a^ determined by Kruskal–Wallis Multiple Comparisons *P* values (2-tailed) between stage 1 and 2; ^c^ determined by Kruskal–Wallis Multiple Comparisons *P* values (2-tailed) between stage 1 and 3; ^#,$^ determined by Mann–Whitney U-test between *cvs*. Drobnica and Buza at the same ripening stage from September to November. Abbreviations: FA—Free acidity (% oleic acid); PV—Peroxide value (mmol O_2_/kg oil).

**Table 3:** Composition of unsaponifiables at various stages of Drobnica and Buza ripening.

	September		October		November	
	DROBNICA (20/09/1999)	BUZA (21/9/1999)	DROBNICA (09/10/1999)	BUZA (14/10/1999)	DROBNICA (3/11/1999)	BUZA (7/11/1999)
Unsaponifiables (%)	1.90 ± 0.09	1.85 ± 0.05	2.58 ± 0.11	1.47 ± 0.08	2.31 ± 0.13	2.37 ± 0.11
Sterols (%)						
Campesterol	4.73 ± 2.75	6.01 ± 0.30	3.24 ± 1.72	2.57 ± 2.44	1.58 ± 1.28	1.88 ± 0.25
Stigmasterol	0.03 ± 0.03	0.02 ± 0.00	0.01 ± 0.01	0.01 ± 0.01	0.11 ± 0.09	0.01 ± 0.01
β-sitosterol ^B^	95.24 ± 2.73	93.95 ± 0.30	96.75 ± 1.71	97.41 ± 2.43	98.27 ± 1.18	98.09 ± 0.25
Total sterols (mg/kg) ^B,D^	2388 ± 444	969 ± 32	2110 ± 97	757 ± 69 ^#^	1354 ± 91	850 ± 94 ^a,$^
α-tocopherol (mg/kg) ^B,D^	154.64 ± 54.66	141.74 ± 7.70	131.71 ± 40.56	131.66 ± 6.13	77.55 ± 5.60	64.97 ± 15.42 ^c,$^
Squalene (mg/kg) ^B,D^	6927 ± 1879	5384 ± 576	9697 ± 299	5641 ± 320 ^#^	5078 ± 1598 ^b^	7696 ± 503 ^c,$^
Alkanols (%)						
nC22 ^B^	8.94 ± 0.12	5.25 ± 0.22	9.05 ± 0.54	8.97 ± 1.33	20.70 ± 6.67	34.54 ± 1.17 ^c^
nC23 ^B,D^	0.10 ± 0.10	0.36 ± 0.30	0.00 ± 0.00	0.13 ± 0.13	2.00 ± 0.13 ^b^	1.56 ± 0.15 ^b^
nC24 ^B,D^	22.42 ± 0.91	14.18 ± 1.00	26.04 ± 0.93	22.51 ± 0.68	48.25 ± 4.34 ^c^	41.42 ± 0.40 ^c^
nC25	0.00 ± 0.00	1.37 ± 1.37	0.00 ± 0.00	1.26 ± 1.26	0.00 ± 0.00	0.00 ± 0.00
nC26 ^B,D^	67.97 ± 1.44	63.14 ± 5.77	48.49 ± 1.10	64.18 ± 2.25	28.75 ± 2.45 ^c^	21.89 ± 0.78 ^b^
nC27	0.58 ± 0.56	0.00 ± 0.00	0.00 ± 0.00	0.41 ± 0.31	0.30 ± 0.02	0.29 ± 0.27
nC28 ^B,D^	0.00 ± 0.00	15.70 ± 4.89 *	16.42 ± 2.57	2.53 ± 2.53 ^#^	0.00 ± 0.00	0.30 ± 0.30 ^c^
Total alkanols (mg/kg) ^B^	204 ± 23	77 ± 11	236 ± 4	43 ± 4	199 ± 26	130 ± 10 ^b^

Note: Data are expressed as mean ± SD (*N* = 3). Different superscripts mark significant difference (*P* < 0.05), as follows: ^B^ determined by Kruskal–Wallis Anova by Ranks for Buza; ^D^ determined by Kruskal–Wallis Anova by Ranks for Drobnica; ^a^ determined by Kruskal–Wallis Multiple Comparisons *P* values (2-tailed) between stage 1 and 2; ^b^ determined by Kruskal–Wallis Multiple Comparisons *P* values (2-tailed) between stage 2 and 3; ^c^ determined by Kruskal–Wallis Multiple Comparisons *P* values (2-tailed) between stage 1 and 3; *^,#,$^ determined by Mann–Whitney U-test between *cvs.* Drobnica and Buza at the same ripening stage from September to November.

**Table 4:** Major phenols in olive oils obtained at various stages of Drobnica and Buza cultivars ripening.

	September		October		November	
	DROBNICA (20/09/1999)	BUZA (21/9/1999)	DROBNICA (09/10/1999)	BUZA (14/10/1999)	DROBNICA (3/11/1999)	BUZA (7/11/1999)
Total phenols (mg/kg GAE) ^B,D^	437.67 ± 10.50	374.98 ± 10.49	316.34 ± 4.70	289.96 ± 9.79	273.26 ± 7.14 ^c^	250.09 ± 5.52 ^c^
DPPH scavenger activity (%) ^B,D^	71.18 ± 0.98	71.48 ± 1.16	59.39 ± 0.75	63.46 ± 0.90	57.31 ± 0.96 ^c^	61.14 ± 0.48 ^c^
HYTY (mg/kg) ^B,D^	2.59 ± 0.04	3.39 ± 0.06	1.98 ± 0.01	2.77 ± 0.14	3.62 ± 0.08 ^b^	3.17 ± 0.07 ^b^
TY (mg/kg) ^B,D^	1.70 ± 0.05	0.78 ± 0.00	0.34 ± 0.04 ^a^	0.69 ± 0.13	0.93 ± 0.08	0.28 ± 0.07 ^c^
OL (mg/kg)	5.76 ± 0.04	4.56 ± 0.11	8.80 ± 0.19 ^a^	6.62 ± 0.06	7.43 ± 0.13	7.49 ± 0.20 ^c^
Lut (mg/kg) ^B^	0.28 ± 0.07	0.48 ± 0.04	2.48 ± 0.09	0.60 ± 0.07	2.62 ± 0.23	1.95 ± 0.08 ^c^
Apig (mg/kg) ^B^	1.60 ± 0.04	0.75 ± 0.01	0.81 ± 0.08	0.78 ± 0.04	1.61 ± 0.08	1.01 ± 0.08 ^c^

Note: Total phenols were determined spectrophotometrically using the Folin–Ciocalteu reagent and expressed as mg/kg gallic acid equivalent of (GAE) the oil. Quantification of individual phenolics was performed by calibration curve using caffeic acid at 280 nm as an external standard. Data are expressed as mean ± SD (*N* = 3). Different superscripts mark significant difference (*P* < 0.05), as follows: ^B^ determined by Kruskal–Wallis Anova by Ranks for Buza; ^D^ determined by Kruskal–Wallis Anova by Ranks for Drobnica; ^a^ determined by Kruskal–Wallis Multiple Comparisons *P* values (2-tailed) between stage 1 and 2; ^b^ determined by Kruskal–Wallis Multiple Comparisons *P* values (2-tailed) between stage 2 and 3; ^c^ determined by Kruskal–Wallis Multiple Comparisons *P* values (2-tailed) between stage 1 and 3. Abbreviations: HYTY—hydroxytyrosol; TY—tyrosol; OL—oleuropein; Lut—luteolin; Apig—apigenin.

**Table 5:** Trace minerals in oil extracted from Drobnica and Buza *cvs*. during fruit ripening.

	September		October		November	
	DROBNICA (20/09/1999)	BUZA (21/9/1999)	DROBNICA (09/10/1999)	BUZA (14/10/1999)	DROBNICA (3/11/1999)	BUZA (7/11/1999)
Na ^B,D^	3.09 ± 0.01	0.43 ± 0.00	0.56 ± 0.10 ^a^	0.14 ± 0.02 ^a^	2.86 ± 0.11	0.32 ± 0.01
K ^D^	4.67 ± 0.18	0.22 ± 0.04	0.52 ± 0.15 ^a^	0.14 ± 0.01	3.08 ± 0.29	0.25 ± 0.23
Ca ^B^	1.81 ± 0.05	4.83 ± 0.00	1.86 ± 0.07	2.31 ± 0.03	2.93 ± 0.12	1.10 ± 0.03 ^c^
Mg ^B,D^	0.98 ± 0.01	0.61 ± 0.00	0.33 ± 0.01 ^a^	0.27 ± 0.01 ^a^	0.74 ± 0.02	0.45 ± 0.01
Fe ^B,D^	2.00 ± 0.30	2.42 ± 0.05	1.00 ± 0.13 ^a^	2.65 ± 0.02	1.69 ± 0.04	1.79 ± 0.03 ^b^
Zn ^B,D^	0.13 ± 0.00	0.58 ± 0.01	0.27 ± 0.00	0.31 ± 0.01 ^a^	0.90 ± 0.00 ^c^	0.46 ± 0.01
Cu ^B,D^	0.97 ± 0.01	0.55 ± 0.00	0.42 ± 0.03	0.31 ± 0.01 ^a^	1.28 ± 0.03 ^b^	0.41 ± 0.00
Pb	n.d.	n.d.	n.d.	n.d.	n.d.	n.d.
Cd	n.d.	n.d.	n.d.	n.d.	n.d.	n.d.
Ni	0.15 ± 0.03	0.10 ± 0.01	0.15 ± 0.05	0.10 ± 0.03	0.22 ± 0.15	0.12 ± 0.01
Cr	n.d.	n.d.	n.d.	n.d.	n.d.	n.d.
Ba	n.d.	n.d.	n.d.	n.d.	n.d.	n.d.
Al ^B,D^	0.64 ± 0.01	0.43 ± 0.02	0.22 ± 0.02	0.14 ± 0.02	0.42 ± 0.03 ^a^	0.55 ± 0.02 ^b^
P ^B,D^	9.03 ± 0.15	4.43 ± 0.15	1.10 ± 0.20	1.13 ± 0.35 ^a^	6.50 ± 0.30 ^a^	2.47 ± 0.35

Note: Data are expressed as mean ± SD (*N* = 3). Different superscripts mark significant difference (*P* < 0.05), as follows: ^B^ determined by Kruskal–Wallis Anova by Ranks for Buza; ^D^ determined by Kruskal–Wallis Anova by Ranks for Drobnica; ^a^ determined by Kruskal–Wallis Multiple Comparisons *P* values (2-tailed) between stage 1 and 2; ^b^ determined by Kruskal–Wallis Multiple Comparisons *P* values (2-tailed) between stage 2 and 3; ^c^ determined by Kruskal–Wallis Multiple Comparisons *P* values (2-tailed) between stage 1 and 3.
